# Socio-ecological dynamics of Caribbean coral reef ecosystems and conservation opinion propagation

**DOI:** 10.1038/s41598-018-20341-0

**Published:** 2018-02-07

**Authors:** Vivek A. Thampi, Madhur Anand, Chris T. Bauch

**Affiliations:** 1 Department of Applied Mathematics, University of Waterloo, Waterloo, N2L 3G1 Canada; 2 School of Environmental Sciences, University of Guelph, Guelph, N1G 2W1 Canada

## Abstract

The Caribbean coral reef ecosystem has experienced a long history of deterioration due to various stressors. For instance, over-fishing of parrotfish – an important grazer of macroalgae that can prevent destructive overgrowth of macroalgae – has threatened reef ecosystems in recent decades and stimulated conservation efforts such as the formation of marine protected areas. Here we develop a mathematical model of coupled socio-ecological interactions between reef dynamics and conservation opinion dynamics to better understand how natural and human factors interact individually and in combination to determine coral reef cover. We find that the coupling opinion and reef systems generates complex dynamics that are difficult to anticipate without use of a model. For instance, instead of converging to a stable state of constant coral cover and conservationist opinion, the system can oscillate between low and high live coral cover as human opinion oscillates in a boom-bust cycle between complacency and concern. Out of various possible parameter manipulations, we also find that raising awareness of coral reef endangerment best avoids counter-productive nonlinear feedbacks and always increases and stabilizes live coral reef cover. In conclusion, an improved understanding of coupled opinion-reef dynamics under anthrogenic stressors is possible using coupled socio-ecological models, and such models should be further researched.

## Introduction

Coral reef ecosystems are complex aquatic systems structurally composed of scleractinian corals situated on the accumulated dead exoskeletons of their ancestors^1^. Coral reefs are host to a diverse combination of organisms while offering a multitude of services to the population surrounding them. Each coral reef consists of the multiple base units–polyps–that over a large period of time develop into large coral reef ecosystems which often act as a magnet for both tourism and fishing.

A key asset for coral growth are zooxanthellae algae, which are unicellular organisms capable of photosynthetic processes. In coral reefs, zooxanthellae algae exist in a symbiotic relationship with coral polyps (although we note that zooxanthellae can also exist in isolation). In order to acquire nutrients, corals secrete a chemical signal causing the zooxanthellae algae in the coral tissue to release organic compounds created during photosynthetic processes^2^. In return, zooxanthellae acquire nutrients such as nitrogen in higher densities via the coral excrement^2^. This mutual feedback cycle promotes growth and development of both species.

**Figure 1:**
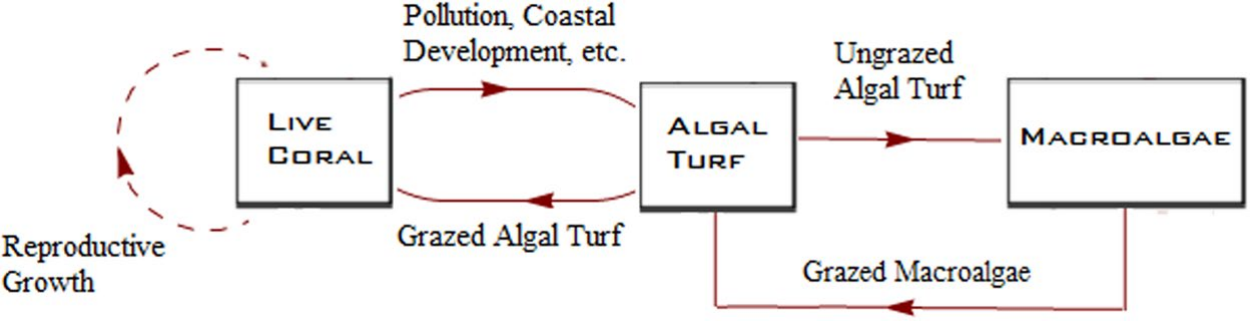
Flowchart illustrating the basic dynamics of the coral reef ecosystem.

Historically, Caribbean coral reefs have been subjected to various stressors, such as coral disease and hurricane-induced destruction^3,4^. Despite the influence of these and other stressors, Caribbean coral reefs have demonstrated considerable resilience against past disturbances. For instance, Caribbean coral reefs began recovering quickly after Hurricane Allen over a 3 year period from 1980 to 1983^3,4^. This resilience is widely attributed to the presence of its dominant grazers, the *Diadema antillarum* sea urchins^3–5^. The mutualistic relationship between the urchins and the coral reefs provided the urchin population with nourishment in the form of algal turf (Fig. 1)^3^. However, the mass mortality of *Diadema antillarum* in 1983, possibly on account of multiple stressors^6^, appears to have reduced the resilience of coral reefs and caused a period of decline in the face of stressors that reefs previously demonstrated resilience against, such as hurricanes^3^. Parrotfish are now considered the primary grazers of the ecosystem, but overfishing of parrotfish has further reduced coral reef resilience, underscoring the need for effective conservation measures^5,7^.

Algal formations are detrimental to the integrity of coral reefs. Invasion of macroalgae is known to prevent reef growth due to their colonization of potential growth sites for new polyps. In the absence of grazing, macroalgal contact with corals have been known to cause higher levels of coral mortality and bleaching^5^. Multiple genera of these algae exist, but for this paper we only consider two species of macroalgae – *Lobophora variegata* whose effects on the reefs induce heightened levels of coral mortality and an extended state of coral bleaching, and *Dictyota* species which cause heightened coral mortality and rapid coral tissue deterioration^8,9^.

Mathematical modelling has been an invaluable tool for many decades, offering insight into real-world systems with applications ranging from classical predator-prey dynamics^10^, to infectious disease dynamics^11^. Relatively detailed mathematical models with empirically-informed model structure and parameter estimates can be useful for predicting future population dynamics under various possible scenarios. However, even simple models can be useful for gaining potential insights into dynamics of ecosystems where the component species interact nonlinearly^12^. Research on coral reef ecosystem dynamics has included mathematical models that explore the impact of various stressors on the coral reefs, including the effect of exploitation^3,13^. Among other findings, these models predict that when predation/fishing of grazers is sufficiently low, the system exhibits a critical transition beyond which the growth and recovery of the ecosystem are possible^3,13^.

Models typically treat human influence on coral reefs as constant and incorporate it through a fixed model parameter, such as fishing intensity. In contrast, coupled socio-ecological models (or equivalently, human-environment system models or coupled human-and-natural system models) formulate a separate dynamical equation for humans, and allow for human systems and ecological systems to influence one another. To capture the effect of dynamic human social behaviour, elements of evolutionary game theory have been previously employed in order to capture social learning dynamics, in which human opinions or strategies actively change based on the current condition of the environment^14^. Humans are assumed to follow an imitation dynamic whereby individuals imitate more successful strategies they observe in other individuals. Models have already been established in ecosystem management^15,16^, forestry/grasslands^16–19^, land-use change^20^ and vaccination^11,14^ but have not been utilized modelling the dynamics of coral reef ecosystems, to our knowledge. For many systems, interactions between humans and natural systems is complex and do not need to be one-way^21^. This approach of coupling human social dynamics to environmental or ecological dynamics contrasts with treating human behaviour as a fixed parameter, as in much previous research on coral reef dynamics, enabling us to model how human opinion about reef conservation and fishing restrictions responds to changes in coral reef cover.

The impact of dynamic human behaviour on coral reef sustainability therefore requires further investigation via theoretical modelling. With evidence of various anthropogenic impacts reflected in current research together with ongoing efforts to promote reef conservation through marine protected areas and other measures^22^, mathematical models of coral reef ecosystem dynamics can benefit from including a coupling to a human population with evolving opinions. By modelling human behavior as an adaptive, dynamically evolving phenomenon, a deeper and richer understanding of the anthropogenic stresses in a given system over longer time horizons–including potential surprises due to nonlinear interactions–can be achieved. With further development, this approach has the potential to assist developing more effective coral reef conservation by helping policymakers avoid counter-productive response by human populations and/or by harnessing processes like social learning to optimize conservation.

Here, we create a coupled socio-ecological coral reef model by combining an existing model of a Caribbean reef ecosystem including parrotfish fishing, with an imitation dynamic model of human opinion spread and behaviour. Our objective is to explore how adaptive human feedback influences the viability of coral reefs, and also to explore the potential dynamics that may emerge in a socio-ecological reef-opinion system that may not be recognized from studying these systems in isolation from one another. We use the model to explore the impact of social norms, sensitivity of the human population to coral loss, and social learning on the coral reef ecosystem. Through these investigations, the effect of social dynamics on the coral reefs are investigated, allowing us to explore potential conditions to improve the long-term viability of the Caribbean coral reef ecosystem.

## Model

We built our socio-ecological model by expanding a previous coral reef ecosystem model by Blackwood *et al*.^13^. We used this model because it is relatively recent and well-documented, and because it is formulated as a system of ordinary differential equations, which facilitates its incorporation into a socio-ecological model based on replicator (imitation dynamic) equations–a type of differential equation model. This model was in turn a modified version of the mathematical model developed by Mumby *et al*.^23^. The latter authors observed a hysteretic feedback loop based on the dynamics of three key components of the ecosystem – macroalgae (*M*), live coral (*C*) and algal turf (*T*). The model was expanded by Blackwood *et al*. to include parrotfish density (*P*)–a key component of the ecosystem–into the model. By incorporating parrotfish grazing dynamics into the model, with loss based on a fixed fishing pressure, the authors were able to determine trajectories for recovery based on the fishing pressure. Originally, Blackwood *et al*. extended the model to include grazing based on the relative density of parrotfish which was affected by the overall fishing pressure in the system^13^.

We extended the Blackwood *et al*. model by adding a fifth equation representing the proportion *x* of the human population currently adopting an opinion in favour of coral reef conservation by reducing parrotfish fishing (we will call these individuals “protectors” for brevity). We consider a human population at the level of local organizations or national populations who can influence decisions about fishery regulation in national waters. The resulting system of five equations for the proportion of macroalgae, live coral, algal turf, parrotfish density, and human opinion is given by:

1 $$\frac{dM}{dt}=aMC-\frac{PM}{M+T}+\gamma MT$$

2 $$\frac{dC}{dt}=rTC-dC-aMC$$

3 $$\frac{dT}{dt}=\frac{PM}{M+T}-\gamma {{\rm M}}{{\rm T}}-rTC+dC$$

4 $$\frac{dP}{dt}=sP(1-\frac{P}{K(C)})-\sigma P(1-x)$$

5 $$\frac{dx}{dt}=\kappa x(1-x)\,(\,-\,1+J(1-C)-{\rm{\sigma }}{P}(1-x)+\varphi (2x-1))$$

The first four equations above are identical to the model of Blackwood *et al*. except that constant parrotfish fishing intensity has been replaced by the coupling term σ *P*(1 − *x*) which reflects the influence of public opinion. We do not explicitly model the mechanism by which public opinion influences fishing intensity, but in real populations it could apply through public pressure and/or special interest group pressure in support of legislation for a marine protected area, for instance. Public opinion (*x*) in turn is influenced by the coral reef cover *C* (the *J*(1 − *C*) term); the economic and social benefits of parrotfish fishing (the σ *P*(1 − *x*) term); a fixed cost of reducing parrotfish fishing through marine protected areas (this is represented by the −1 term since the *x* equation has already been rescaled in the above equations; see Methods for details); and the effects of injunctive social norms (the *ϕ* (2*x* − 1) term). The parameter *ϕ* represents the strength of injunctive social norms. Injunctive social norms represent the social pressure that an individual adopting a minority opinion feels from those with majority opinions, on issues with an ethical or moral dimension.

The model assumes that macroalgae overgrow coral at a rate of *a* per year and spread over ungrazed algal turf over a rate of *γ* per year. In addition, corals overgrow grazed algal turf at a rate of *r* per year and have a natural mortality of *d* per year. Since parrotfish have become the primary grazers in these ecosystems^5,24^, the model focuses on parrotfish population dynamics, grazing and fishing. The model assumes logistic growth of the parrotfish population, proportional to the amount of algal turf grazed, with a growth rate of *s* per year; a carrying capacity term reflective of the current proportion of live coral, *K*(*C*); and a fishing rate based on the number of protectors, σ (1 − *x*)^13^, in the population where x represents the proportion of protectors in the population and 1 − *x* represents the proportion of the population opting to continue fishing and, effectively, increase the total fishing pressure. Details pertaining to the development of the model and the human dynamic, and the definition of the parameters are located in the Methods section.

**Figure 2:**
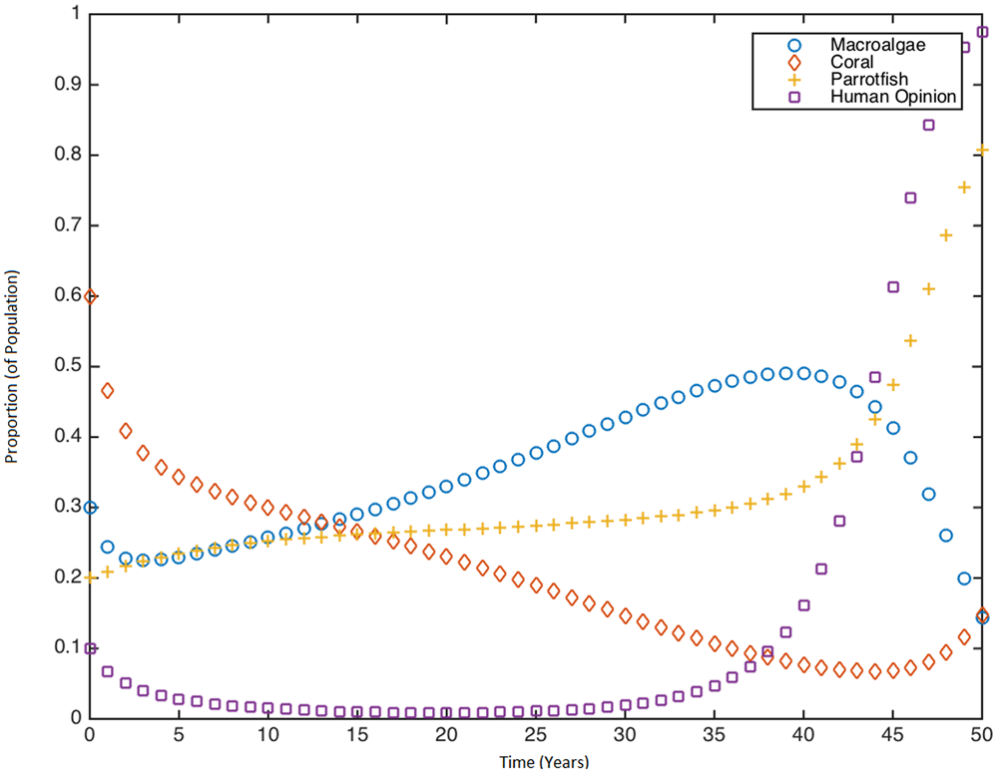
Time series of reef-opinion dynamics at baseline parameter values, showing response of the human population to declining coral reef cover. Baseline parameter values are *κ* = 1.014 *yr*^−1^, *J* = 1.68, *ϕ* = 0.2. Initial conditions are *M*(0) = 0.3, *C*(0) = 0.6, *P*(0) = 0.2, *x*(0) = 0.1.

Our model used the same baseline parameter values for the coral reef component as the model of Blackwood *et al*. and Mumby *et al*.^3,13^, and have been included in Table 1 of Appendix A of the supplementary information file. In particular, we assumed the rate of macroalgal overgrowth of corals, *a* = 0.1 *yr*^−1^, the rate of macroalgal growth over ungrazed algal turf, *γ* = 0.8 *yr*^−1^, the rate of coral growth over grazed algal turf *r* = 1 *yr*^−1^, coral mortality rate, *d* = 0.44 *yr*^−1^, and the parrotfish growth rate, *s* = 0.49 *yr*^−1^, consistent with the previous models. The parameter observing human-induced parrotfish mortality σ can vary over a range of [0, 1], but baseline was assumed at 0.5 *yr*^−1^. Values for *κ*, *J* and *ϕ* were calibrated to yield biologically and sociologically plausible behaviour as follows. Coral reefs associated with various Caribbean islands were impacted differently by stressors. However, overall live coral cover declined significantly across the Caribbean region over several decades, causing macroalgal formations to dominate many Caribbean coral reef ecosystems^5^. This in turn stimulated conservationism and a demand for marine protected areas^23^. Hence, we sought values of *κ*, *J* and *ϕ* such that coral cover declines from an initially high level to be replaced by growing macroalgal turf due to parrotfish over-fishing, which in turns stimulates a growth in protector opinion that restrains parrotfish fishing and stabilizes coral cover. The resulting model trajectory for coral reef cover from *t* = 5 to *t* = 45 years (Fig. 2) is qualitatively similar to the decline in coral reef cover reported by Jackson *et al*. over three successive time intervals (1969–1983, 1984–1998 and 1999–2011) from 34.8% to 19.1% to 16.3%^5^. Longitudinal data on coral reef conservation opinions in Caribbean populations are not available, so as a proxy we used longitudinal data on conservation opinions on a range of issues in the United States from 1965 to 1990, a time period corresponding to a significant shift in attitudes regarding conservation. Our baseline change in coral reef conservation opinion from *t* = 20 to *t* = 45 years (Fig. 2) was likewise calibrated to the observed changes in the United States data.

Model simulations at the baseline parameter values follow a trajectory of live coral cover similar to that observed in populations where coral decline has stimulated successful conservationism (Fig. 2)^5^. Parrotfish start out initially low due to overfishing and lack of conservationism, which causes a gradual decline in coral cover. However, when coral cover gets too low, human opinion shifts in favour of conservationism.

## Results

**Figure 3:**
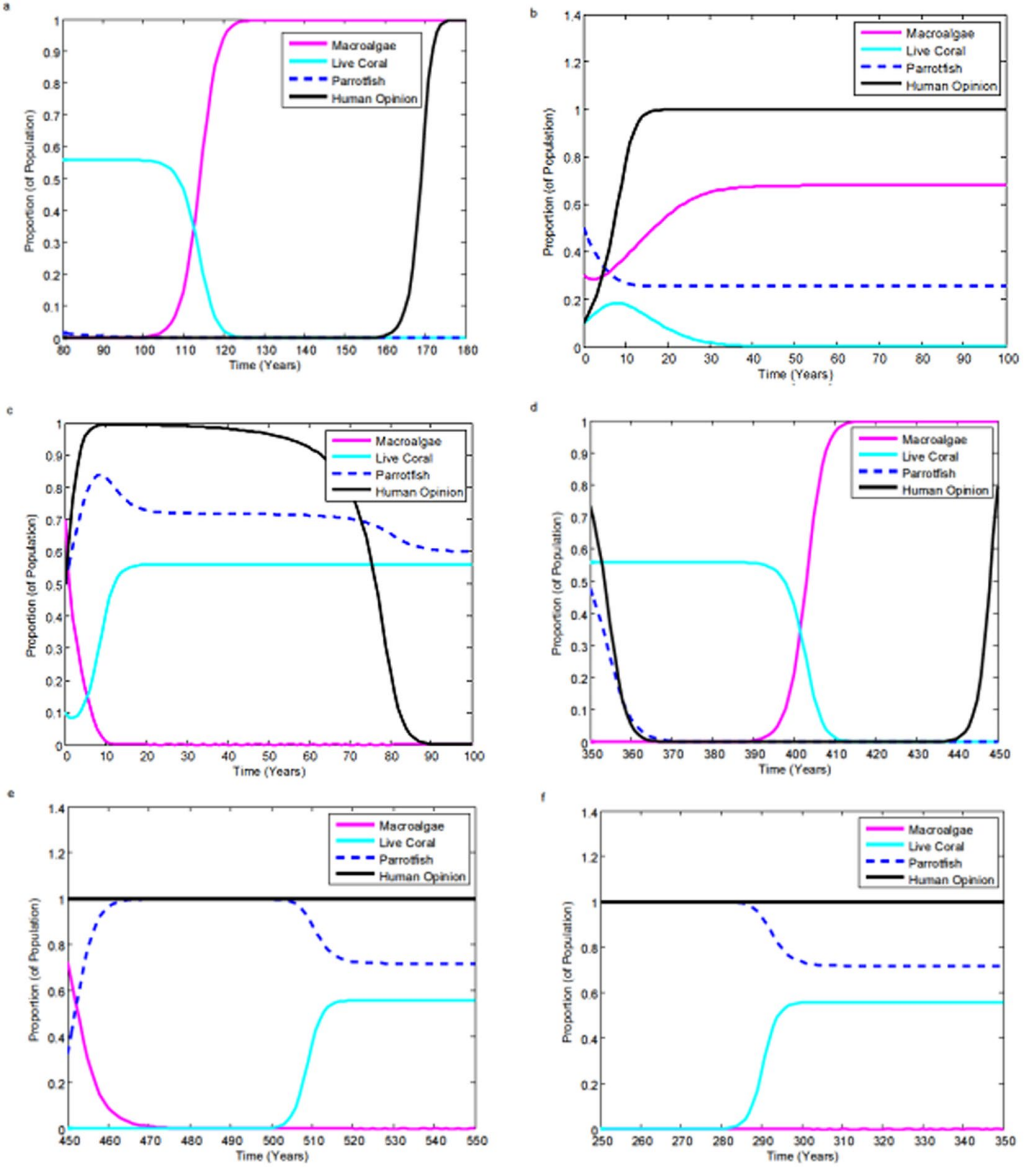
Time series depicting: (**a**) The catastrophic equilibrium with complete parrotfish eradication, where *s* = 0 *yr*^**−**1^, and σ = 0.1 *yr*^−1^. (**b**) The catastrophic equilibrium with parrotfish survival, where *s* = 0 *yr*^−1^, and σ = 0.15 *yr*^−1^. (**c**) The unassisted macroalgae-free equilibrium, where *s* = 0.3 *yr*^−1^, and σ = 0.05 *yr*^−1^. (**d**) Oscillatory behaviour, where *s* = 0.2 *yr*^−1^, and σ = 0.5 *yr*^−1^. (**e**) Oscillatory behaviour which eventually converges to the assisted macroalgae-free equilibrium, where *s* = σ = 0.4 *yr*^−1^. (**f**) The assisted macroalgae-free equilibrium, *s* = 0.35 *yr*^−1^, and σ = 0.5 *yr*^−1^. All other parameters remain at baseline values.

Time series of the model dynamics across a selection of parameter values illustrate the range of possible dynamics of this socio-ecological system. For instance, when the parrotfish growth rate *s* is zero (Fig. 3a,b), dependent on the initial conditions, the system enters an undesirable equilibrium state of high macroalgal cover and no live coral. Conservationism is strong, but because the parrotfish population is not viable, the coral reefs cannot survive. However, under parameter conditions where parrotfish growth rate sufficiently outweighs the rate of parrotfish exploitation σ, the system is predicted to recover and achieve stability. The system stabilizes as a ‘Macroalgae-Free Equilibrium’ (MFE). This can occur with human assistance via marine protected areas as in the baseline scenario - MFE-A (*s* = 0.35, σ = 0.5), (Fig. 3(f)) or without anthropogenic assistance, if the fishing rate even at maximal levels is not enough to overcome the parrotfish growth rate even in the absence of marine protected areas - MFE-NA (*s* = 0.3, σ = 0.05), Fig. 3(c)).

It is also possible for the model to exhibit oscillations over long time horizons. During periods of sufficient conservationism in the human population, the system successfully recovers. During periods of sustained recovery, coral reef cover becomes high again, which means that parrotfish exploitation becomes attractive compared to continued conservationism (the human popula-tion becomes complacent about conservationism). As a result, conservation eventually becomes less popular and coral reefs correspondingly begin to decline again (*s* = 0.5, σ = 0.2) (Fig. 3(d)). In some cases, this oscillatory behaviour is predicted to wane, and the utility to protect permanently outweighs the utility to not protect, yielding an MFE-A over the long term (Fig. 3(e)). Additional time series depicting other, less common dynamical regimes of the model appear in Appendix B of the supplementary information file, including variations of the catastrophic equilibrium for parameter values away from baseline values.

To explore model dynamics more systematically we generated a series of parameter planes. A parameter plane shows how model dynamics vary under changes in two different model parameters. The plane has a model parameter on each axis, and indicates the dynamics that occur for each possible pair of parameter values. For each parameter plane, all parameters were held at their baseline tables except for the two parameter values being varied in the plot. Using Matlab, we varied the values of the selected parameters across the range depicted in the parameter planes. Time series were generated for each pair of values under a range of initial conditions for *M*, *C*, *P* and *x*, and their long-term states were identified. Gnuplot was used to render the parameter planes.

Using the range of parrotfish population doubling time reported in FishBase^25^, we derived a range of realistic growth rate values *s* from 0.16/year to 0.50/year. Hence, parameter planes varying *s* were taken to range from 0/year to 0.6/year. Fishing rates are highly variable and since our objective was to gain qualitative insights, we chose the fishing rates to capture the full range of possible outcomes from very little fishing to fish population collapse. Population collapse in the model occurs when the maximal fishing rate exceeds the population growth rates (if conservationist opinion is weak and the fish population is low). Hence, the range for the maximal fishing rate σ also runs from 0/year to 0.6/year. Ranges for *κ*, *J* and *ϕ* were chosen to include enough representation of values on either side of the baseline value to capture a variety of possible dynamics.

The dynamical regimes we discovered through this process are summarized in Table 1 where they are divided into three primary regimes corresponding to healthy coral reefs without the need for human assistance (which is the least realistic scenario, given modern-day maximal fishing intensities); healthy coral reefs with human assistance; and dead corals. Other less common dynamical regimes are described in Table 2 as well.

**Table 1:** Parameter plane regions and descriptions for healthy corals and dead corals.

Classification	Description
Healthy Corals - Unassisted	The most desired result. The system follows a trajectory which stabilizes in the regime governed by the human - unassisted macroalgae-free equilibrium (MFE-NA). Achieving stability within this regime implies conservation does not need to be permanently employed. The system will preserve its resilient state despite individuals not opting to promote conservation, minimizing the total costs and achieving the most desirable state of the ecosystem.
Healthy Corals - Assisted	Another desired result. Similar to the MFE-NA, under certain initial conditions the system will stabilize to the human-assisted macroalgae-free equilibrium (MFE-A). Contrary to the Healthy Corals - MFE-A, the system is incapable of maintaining its resilience without conservation. Despite the additional costs incurred to stabilize the system, this is still a desirable state as the recovery of the ecosystem has successfully been achieved, increasing attractiveness for tourism.
Dead Corals	The most undesirable result. The system converges to a state dominated by macroalgae and dead coral. The system admits 4 different variations of this result: (**i**) A bistable regime where the system converges either to a macroalgal dominant state with complete conservation, living parrotfish and coral death or a macroalgal dominant state with complete conservation, parrotfish death and coral death. (**ii**) Convergence to a state of complete coral death with complete macroalgal overgrowth and live parrotfish density without conservation. (**iii**) Convergence to state of complete coral and parrotfish death and complete macroalgal overgrowth without conservation. (**iv**) Convergence to a state devoid of macroalgae and corals, with conservation practices in motion and live parrotfish density (shown in Appendix B of the supplementary information file).

**Table 2:** Additional parameter plane regions.

Region	Behaviour
A	Bistable regime, converging to either a coral death equilibrium with sustainable parrotfish density or the unassisted macroalgae-free equilibrium.
B	Behaviour stabilizes to either a limit cycle or the assisted macroalgae-free equilibrium.
C	Tristable regime, stabilizing to either: (**1**) a coral death equilibrium with parrotfish survival and full conservation cooperation, (**2**) a catastrophic equilibrium with parrotfish extinction with full conservation cooperation or (**3**) the assisted macroalgae-free equilibrium.
D	Bistable regime, stabilizing to either the assisted macroalgae-free equilibrium or a state of coral and macroalgal decimation, with human-assisted parrotfish survival.
E	Tristable regime, stabilizing to either: (**1**) the unassisted macroalgae-free equilibrium, (**2**) the assisted macroalgae-free equilibrium, or (**3**) a state governed by a human-unassisted coral death equilibrium, with sustainable parrotfish density.
F	Bistable regime, stabilizing to either the unassisted macroalgae-free equilibrium or a state governed by the human-unassisted coral death equilibrium, with sustainable parrotfish density.
G	Tristable regime, with behaviour converging to either behaviour of (**D**), or a state governed by the human-unassisted coral death equilibrium, solely governed by macroalgae.
H	Convergence to limit cycles.

**Figure 4:**
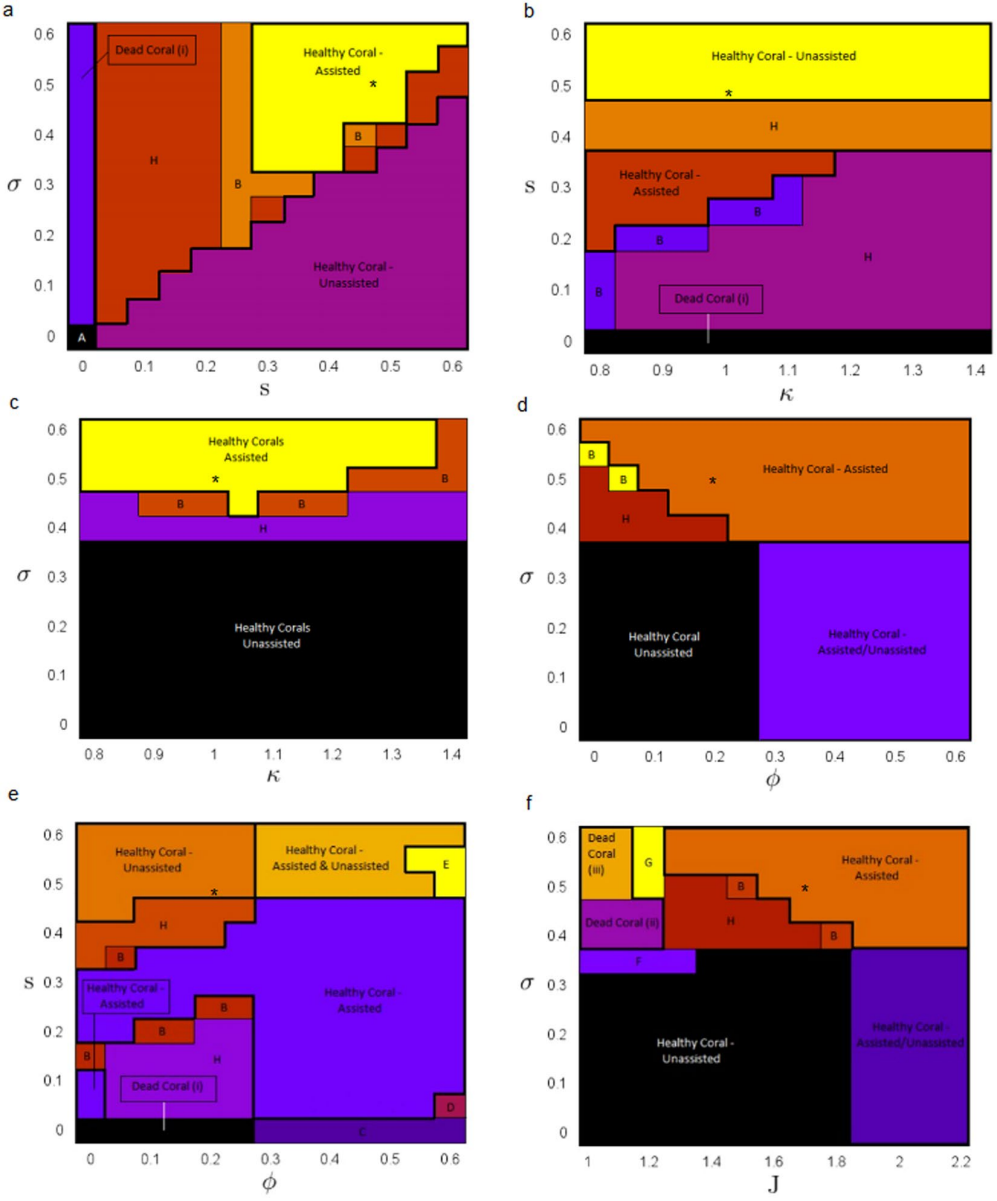
Parameter planes depicting the model dynamical regimes under parameter variations in, (**a**) the parrotfish growth rate *s* and the maximal fishing rate σ, with all other parameters held at baseline values, (**b**) the social learning rate *κ* and the parrotfish growth rate *s*, with all other parameters held at baseline values, (**c**) the social learning rate *κ* and the maximal fishing rate σ, with all other parameters held at baseline values, (**d**) the effect of social pressure *ϕ* and the maximal fishing rate σ, with all other parameters held at baseline values, (**e**) the effect of social pressure *ϕ* and the parrotfish growth rate *s*, with all other parameters held at baseline values, and (**f**) the coral density sensitivity *J* and the maximal fishing rate σ, with all other parameters held at baseline values. *Denotes the baseline value of the parameter. See Tables 1 and 2 for the interpretation of the dynamical regimes.

Model dynamics under variation in the maximal fishing rate s and the parrotfish growth rate s depend on the relative size of these two competing parameters (Fig. 4(a)). When the growth rate exceeds the fishing rate, then the coral is able to maintain itself in a healthy state without human influence, and the system follows a trajectory stabilizing to the unassisted macroalgae-free equilibrium (MFE). However, when the fishing rate exceeds the growth rate, a number of outcomes are possible. For instance, when the growth rate is too low, the coral simply dies off completely, or the system oscillates in all of its variables (regime H). When the parrotfish growth rate is higher, it is possible for coral to persist in a healthy state with human assistance, but other dynamical regimes also occur in this part of parameter space, such as oscillations once again (regime H, B). Finally, regardless of the fishing rate, when the growth rate is too small (*s* ≈ 0), the corals collapse along with the parrotfish population. If the fishing rate σ is also too small, the final state of the ecosystem becomes solely dependent on the initial state, converging to the bistable regime.

Under varying levels of the social learning rate *κ*, the model dynamics indicated strong dependence on the relative sizes of the maximal fishing rate σ and the parrotfish growth rate *s*. At the baseline value of the fishing rate, when the parrotfish growth rate is sufficiently high, coral is able to follow a trajectory towards the unassisted macroalgae-free equilibrium (Fig. 4(b)). Conversely, at the baseline value of the growth rate, low fishing rates cause the system to follow a trajectory to the unassisted macroalgae-free equilibrium (Fig. 4(c)). In the absence of growth, the model predicts coral collapse (*Dead coral (i)*), regardless of the initial conditions of the ecosystem. Alternatively, at the baseline growth rate, a significant increase in maximal fishing intensity causes the ecosystem to transition from unassisted macroalgae-free equilibrium to assisted macroalgae-free equilibrium (Fig. 4(c)). In addition, if the system exhibits small (but not too small) parrotfish growth, it does not predict coral collapse. So long as growth is present, conservation causes the ecosystem to adopt an oscillatory trajectory (regime H) or the MFE-A equilibrium (Fig. 4(b)).

Variation of the strength of injunctive social norms *ϕ* revealed its impact on model dynamics in the context of varying the maximal fishing rate σ (Fig. 4(d)) and parrotfish growth rate *s* (Fig. 4(e)). At baseline parrotfish growth, when the maximal fishing rate sufficiently overwhelms the strength of social norms, corals are able to survive under human assistance, following a trajectory towards the assisted macroalgae-free equilibrium (Fig. 4(d)). This occurs because social norms in support of conservationism can maintain the popularity of conservationism, as the population tends to conform to the majority opinion. When fishing rates are lower, then the coral is able to survive either with or without human assistance, depending on the value of *ϕ*. Alternatively, at baseline fishing rates, a wide variety of possible outcomes are observed for various values of the strength of social norms *ϕ* and the growth rate *s* (Fig. 4(e)). For sufficiently low strength of social norms, the corals maintain a trajectory towards the unassisted macroalgae-free equilibrium. As the strength of social norms increases, the model predicts ecosystem dynamics to shift towards a bistable regime governed by either the assisted or unassisted macroalgae-free equilibrium. This occurs because social norms can also operate to suppress conservationism, if it was not sufficiently popular at first in the population. This effect can be clearly observed where a significant increase in the strength of social norms at sufficiently high levels of parrotfish growth (but not maximal growth) can force the system into a tristable state, following a trajectory towards either macroalgae-free equilibrium, or towards a catastrophic state with live parrotfish density (Regime E). The opposite effect can observed under periods of low parrotfish growth. Under certain initial conditions, social norms can operate to enhance conservationism, causing ecosystem dynamics to shift towards the assisted macroalgae-free equilibrium. Finally, for sufficiently effective social norms, and sufficiently low parrotfish growth, corals are still able to thrive with human support. If social norms become too influential, the ecosystem can either maintain its coral integrity, or be driven into a state completely devoid of macroalgae or corals, with efforts in conservation protecting the parrotfish community (Regime D), but unable to restore corals.

Model dynamics under variation in human sensitivity to coral reef rarity *J* and the maximal fishing rate σ depend on the relative sizes of these two parameters, and dynamics again display a complicated dependence on the parameter values (Fig. 4(f)). When the fishing rate and sensitivity terms are sufficiently low, corals are able to dominate and the ecosystem converges to the unassisted macroalgae-free equilibrium. As the maximal fishing rate increases, the system produces different behaviour. For sufficiently small sensitivity, corals die out and the ecosystem follows a trajectory to either a catastrophic equilibrium with living parrotfish and no protection *(Dead coral (ii))* or the catastrophic equilibrium with no parrotfish, and no protection *(Dead coral (iii)*. For sufficiently high sensitivity, corals are able to sustain themselves in a healthy state due to human intervention, and the ecosystem stabilizes at the assisted macroalgae-free equilibrium. Lastly, for sufficiently low fishing rates and sufficiently high sensitivity, the system transitions into a bistable regime governed by both macroalgae-free equilibria. High sensitivity can force the population to adopt conservationism, despite the resilient state of the coral reefs. Depending on the initial state, efforts in conservation can become perpetually enforced, driving the system to converge to the assisted macroalgae-free equilibrium.

## Discussion

We coupled an existing model of Caribbean coral reef ecosystem dynamics to a model of human opinion dynamics. The resulting coupled socio-ecological model was used to explore how coral reef viability depends on nonlinear interactions between social and ecological factors individually and in combination, when opinion dynamics and reef dynamics are coupled to one another. To our knowledge, this is the first mathematical model to capture coupled socio-ecological dynamics of a coral reef ecosystem. Our model suggests that increasing human sensitivity to declining coral reef cover (for instance, through awareness programs) is the best way to support live coral and parrotfish densities. This model result is consistent with empirical findings that increased awareness of coral decline can help stimulate coral reef protection^26^.

The added benefit of developing models in addition to using empirical studies is that models can help develop intuition for nonlinear feedbacks; test the logic of existing hypotheses that concern nonlinear feedback mechanisms; and suggest new ideas and new hypotheses. Here, we found that higher levels of injunctive social norms (i.e. social pressure) can also support conservationism, if the initial state of the population and other natural and economic factors already tend to support it. However, sometimes it can also be a double-edged sword since conservationism can be suppressed due to social pressure if it is not initially a dominant opinion. The system also tended to oscillate in boom-bust cycles, as human populations become complacent when coral reefs are robust, but react to support conservationism when they become endangered again. This is especially common for higher rates of social learning. This exemplifies a predicted dynamic that could not be identified without constructing a nonlinear mathematical model. Finally, the harmful effects of increasing the maximal fishing intensity were often partially counteracted by the conservationist response that higher fishing intensities stimulated in the population, such that conservationism was able to help sustain live coral cover even at high maximal fishing rates under some conditions.

Results of sensitivity analyses revealed trends similar to that of earlier models developed by Mumby *et al*. and Blackwod *et al*.^3,13^. When grazing is at a minimum, the models predict convergence to an equilibrium of algal overgrowth. Alternatively, when grazing is sufficiently large and critical fishing thresholds are not exceeded, all three models predict convergence to a desirable, coral-dominated state (the MFE-A is admitted in our case). In comparison to the previous ecological models, by coupling a human dynamic to the model, we have achieved a novel condition for recovery whereby both, fisheries and corals are capable of thriving and coexistence is achieved (MFE-NA).

It is well-known that Caribbean coral reefs have suffered an extreme decline over the past few decades^5^. Declines in the parrotfish population have already been identified as a core concern, but additional stressors have also been identified, such as sedimentation and coral bleaching^3,4^. The Caribbean coral reef ecosystem has shown little to no resilience since the decline of the *Diadema antillarum* sea urchins despite the implementation of conservational strategies. This is not to say that conservation has not been fruitful, as it has been documented that within smaller sanctuaries, live coral cover has greatly increased due to conservation efforts such as marine protected areas (MPAs), compared to unprotected areas that can be targeted by fisheries^27,28^. Thus, there must not only be a stronger focus on parrotfish protection policies, but also, we require an increase in overall awareness of the coral reef ecosystem and incentives to protect in order to promote the recovery of the Caribbean coral reefs^29,30^. Our results agree with these proposed actions that increasing awareness and concern for declining coral reef cover in the general population might be the most effective and feasible way to do this.

The model assumed only two types of macroalgal growth that are the preferred food source of the dominant grazer but other algal growths have been documented in the reef ecosystem. Multi-species structure can influence model predictions. Hence, future models could include multiple species of algal grazers with differing effects on coral cover, different life histories, and different fishing rates. In the context of Caribbean coral reefs, was noticed by Mantyka and Bellwood in 2007^31^ that parrotfish specialize in the consumption of calcified macroalgae, whereas another local grazer–siganids–were typically much more selective. Experimental results by Burkepile and Hay^27^ illustrated the advantage of species diversity on herbivory, where a combination of parrotfish and surgeon fish can produce a stronger positive response on reef recovery compared to an ecosystem comprising solely of parrotfish. Specifically, within a ten month period, it was discovered that regions with greater herbivorous fish diversity increased coral cover by over 20%, whereas regions maintained by one or less herbivorous fish experienced declines in coral cover of up to 30%. The current model can improved to incorporate this diversity.

It is a common practice in theoretical biology to start with simple models and progress to more complex models over time^12^. The reasons are that (1) simpler models are easier to analyze and require less data to develop, (2) in many situations, simpler models can provide accurate predictions even though they do not include all the details of the system being modelled, and (3) lessons learned in developing the simpler model are helpful when developing more complex models that are harder to analyze and understand. However, simplifying assumptions can influence model predictions and must be highlighted for addressing in future models. For coral reefs, there is limited availability of long-term coral cover data, so the primary use of our model was to generate insights into possible nonlinear feedbacks, suggest new hypotheses and topics for further empirical research, and test the logical validity of hypotheses. For instance, a survey that compares the effects of social learning rates (rate at which individuals read or talk about coral reefs); injunctive social norms; and sensitivity to the amount of coral reef cover (i.e. how concerned individuals become as coral becomes rarer) on attitudes toward coral reef conservation might help refine the model. Not all stressors that currently affect the reefs have been explicitly incorporated into the model, such as sediment contamination. Similarly, the model does not explicitly account for climate change (coral bleaching) in the sense that the modelled human population does not respond adaptively to coral bleaching to take steps to prevent climate change. This was excluded as a state variable because the local and national populations we are modelling can influence parrotfish fishing in their waters, but cannot take effective unilateral action on climate change. However, climate change effects can be captured partially and implicitly in our model by varying parameters such as the coral death rate, *d*. In the future, more sophisticated models could include specific climate change aspects such as interactions between multiple stressors like coral bleaching and growth of algal turf. Other extensions could include spatial structure, multi-species interactions, Allee effects, and more sophisticated fishery dynamics.

While opportunities for future research are clearly numerous, the incorporation of human-environment feedback into a coral reef model showed how ecological and human factors act both individually and in combination to determine coral reef health, and also showed that these interactions can be surprising and nontrivial. Overfishing and reef degradation persists, despite efforts to mitigate them^32^, hence more research on the socio-ecological of coral reef ecosystems and public opinion is urgently needed. Such opportunities to expand this research offer great potential for generating biologically relevant results that can reshape and influence the dialogue around coral and parrotfish conservation in the Caribbean and elsewhere. Hence the model has generated compelling evidence that theoretical approaches to understanding socio-ecological interactions would be helpful in the efforts to restore coral reefs to their former, resilient status.

## Methods

A previously established compartmental model by Blackwood *et al*.^13^ was extended to a coupled human-environment system. The original model is represented by the following system of differential equations:

6 $$\frac{dM}{dt}=aMC-\frac{PM}{M+T}+\gamma MT$$

7 $$\frac{dC}{dt}=rTC-dC-aMC$$

8 $$\frac{dT}{dt}=\frac{PM}{M+T}+\gamma {{\rm M}}{{\rm T}}-rTC+dC$$

9 $$\frac{dP}{dt}=sP(1-\frac{P}{K(C)})-fP$$

where *M*, *C* and *T* represent the proportion of the population of macroalgae, coral and algal turf respectively, and *P* represents the population of parrotfish in the reefs, scaled relative to their carrying capacity. The model admits an induced yearly mortality rate of *f* attributed to exploitation^13^. Values for the parameters were obtained from other studies focused in the Leeward Islands, Central America and the Southern Caribbean^3^.

The effect of human influence in the original model is represented by the fixed parameter *f*. However, human opinion is as dynamic as ecosystem dynamics and can change based on the various policies and laws implemented in order to promote conservation, as well as by responding to coral reef endangerment. Thus, in order to introduce human strategies into the model, we introduce a human-behavioural differential equation in the system.

It is well-known that the coral reefs are highly regarded as a tourist attraction^33,34^. As their condition diminishes, the reef loses value, both ecologically and economically. In order to increase their utility, conservation strategies can be implemented to promote recovery and increase the value of the resource^35^. To generate the basic human behaviour dynamic, utility equations are developed, modelling individuals residing in the above regions, adopting either the”protector” strategy or the”exploiter” strategy.

Similar to vaccination dynamics, we use utility functions to quantify human preferences^11^. We consider a population making decisions about whether to exploit parrotfish or protect the coral reef. Let *U*_*P*_ represent the perceived utility for protecting coral and *U*_*N*_ represent the perceived utility for not protecting coral (i.e. parrotfish exploitation). Let *x* represent the proportion of the population who support coral protection. Consequently, (1 − *x*) represents the proportion of the population who do not want to protect the coral reefs (and, thus support parrotfish fishing). We assume that the utility function for protectors is given by:

10 $${U}_{P}=-\,q+m(1-C)+\delta x,$$

where *q* represents the cost to protect coral (for instance, the cost to set up and monitor a protected area); *m* is a proportionality constant that controls the sensitivity of the protector utility to coral density *C*; and *δ* controls the strength of injunctive social norms, as in similar behavioural models^11^. This utility function captures how the utility (or motivation) for protection increases as coral *C* becomes rare.

Similarly, the utility function for non-protectors is given by:

11 $${U}_{N}={\rm{\sigma }}P(1-x)+\delta (1-x),$$

where σ is the maximal fishing rate and *P* is the proportion of parrotfish in the ecosystem. This utility function captures how the utility of parrotfish exploitation is higher when more parrotfish are exploited, however, a high proportion of protectors in the populations will reduce exploitation.

When *U*_*P*_ − *U*_*N*_ > 0 coral conservation is a much more valuable strategy, whereas if *U*_*N*_ − *U*_*P*_ > 0, parrotfish exploitation is preferred. Let *k* represent the time rate at which individuals sample others in the population^14^. If *U*_*P*_ − *U*_*N*_ > 0, then the rate at which non-protectors switch to a protector strategy is given by

12 $$\frac{dx}{dt}=(1-x)kx({U}_{P}-{U}_{N}).$$

and if *U*_*P*_ − *U*_*N*_ ≤ 0 then non-protectors never switch. Alternatively, if −(*U*_*P*_ − *U*_*N*_) > 0, then the equation of motion is represented by:

13 $$\frac{dx}{dt}=-\,kx(1-x)\,({U}_{N}-{U}_{P}),$$

and if *U*_*P*_ − *U*_*N*_ ≤ 0 then protectors never switch. We sum these two processes to get the total rate of change of *x*:

14 $$\frac{dx}{dt}=kx(1-x)\,({U}_{P}-{U}_{N}).$$

Let *κ* = *kq*, *J* = $\tfrac{m}{q}$ and *ϕ* = $\tfrac{\delta }{q}$ be the rescaled social learning rate, sensitivity term and strength of injunctive social norms respectively. The human behaviour equation thus becomes:

15 $$\frac{dx}{dt}=kx(1-x)\,(\,-\,1+J(1-C)-{\rm{\sigma }}P(1-x)+\varphi (2x-1)).$$

In addition to incorporating a human behaviour model, the compartment describing the dynamics of the parrotfish density must also be modified so that parrotfish exploitation slows down when conservationists are more dominant. This results in the system:

16 $$\frac{dM}{dt}=aMC-\frac{PM}{M+T}+\gamma MT$$

17 $$\frac{dC}{dt}=rTC-dC-aMC$$

18 $$\frac{dT}{dt}=\frac{PM}{M+T}-\gamma {{\rm M}}{{\rm T}}-rTC+dC$$

19 $$\frac{dP}{dt}=sP(1-\frac{P}{K(C)})-\sigma P(1-x)$$

20 $$\frac{dx}{dt}=\kappa x(1-x)\,(\,-\,1+J(1-C)-{\rm{\sigma }}{P}(1-x)+\varphi (2x-1))$$

Note that the death rate term is omitted from the parrotfish dynamics model as its effect is inherited by the loss rate due to predation.

## Electronic supplementary material

Supplementary Information
