# Author Correction: Socio-ecological dynamics of Caribbean coral reef ecosystems and conservation opinion propagation

**DOI:** 10.1038/s41598-018-33573-x

**Published:** 2018-11-08

**Authors:** Vivek A. Thampi, Madhur Anand, Chris T. Bauch

**Affiliations:** 1 Department of Applied Mathematics, University of Waterloo, Waterloo, N2L 3G1 Canada; 2 School of Environmental Sciences, University of Guelph, Guelph, N1G 2W1 Canada

Correction to: *Scientific Reports* 10.1038/s41598-018-20341-0, published online 07 February 2018

The original version of this Article contained errors.

Affiliation 2 was incorrectly given as ‘School of Environmental Science, University of Guelph, Guelph, N1G 2W1, Canada’. The correct affiliation is listed below:

School of Environmental Sciences, University of Guelph, Guelph, N1G 2W1, Canada

An affiliation for Vivek A. Thampi was omitted. The correct affiliations for Vivek A. Thampi are listed below:

Department of Applied Mathematics, University of Waterloo, Waterloo, N2L 3G1, Canada

School of Environmental Sciences, University of Guelph, Guelph, N1G 2W1, Canada

Chris T. Bauch was incorrectly affiliated with ‘School of Environmental Sciences, University of Guelph, Guelph, N1G 2W1, Canada’. The correct affiliation is ‘Department of Applied Mathematics, University of Waterloo, Waterloo, N2L 3G1, Canada’.

Madhur Anand was incorrectly affiliated with ‘Department of Applied Mathematics, University of Waterloo, Waterloo, N2L 3G1, Canada’. The correct affiliation is ‘School of Environmental Sciences, University of Guelph, Guelph, N1G 2W1, Canada’.

In addition, the email addresses for Chris T. Bauch and Madhur Anand, were incorrectly listed as manand@uogelph.ca and cbauch@uwaterloo.ca respectively.

These errors have now been corrected in the PDF and HTML versions of the Article.

