# Supplementary material for: Socio-ecological dynamics of Caribbean coral reef ecosystems and conservation opinion propagation
Source: Sci Rep. 2018 Feb 7;8:2597. doi: 10.1038/s41598-018-20341-0 (PMC5803238; doi:10.1038/s41598-018-20341-0)
Supplement: Supplementary file 1 — Supplementary Information [file 41598_2018_20341_MOESM1_ESM.docx]

Socio-ecological dynamics of Caribbean coral reef ecosystems and conservation opinion propagation Supplementary Information

Vivek A. Thampi, Madhur Anand, Chris T. Bauch

January 10, 2018

# Appendix A - Parameter Values

| Parameter | Definition | Value(s) | Units | Source |
| --- | --- | --- | --- | --- |
| a | rate of macroalgal overgrowth over corals | 0.1 | *year*^−1^ | [? ] |
| *γ* | rate macroalgal growth over ungrazed algal turfs | 0.8 | *year*^−1^ | [? ] |
| r | rate of coral growth over grazed algal turf | 1 | *year*^−1^ | [? ] |
| d | coral mortality rate | 0.44 | *year*^−1^ | [? ] |
| s | growth rate of parrotfish | 0.49 | *year*^−1^ | [? ] |
| *K*(*C*) | non-dimensional term which  limits the carrying capacity of parrotfish as a function of coral  cover | – | – | [? ] |
| *σ* | maximum parrotfish mortality rate due to overfishing | 0.5 (0 ≤*σ* ≤ 1) | *year*^−1^ | [? ] |
| *κ*(= *kq*) | the product of the combined  imitation rate at which people  sample others *k* and switch  strategies, and the cost of adopting a protector strategy *q* | 1.014 | *year*^−1^ | calibrated |
| J | sensitivity of humans to current density of coral cover | 1.68 | – | calibrated |
| *φ* | The adjusted strength of injunctive social norms | 0.2 | – | calibrated |

Table 1: Table of parameters along with their baseline values and sources

# Appendix B - Alternative Non-Baseline Parameter Results


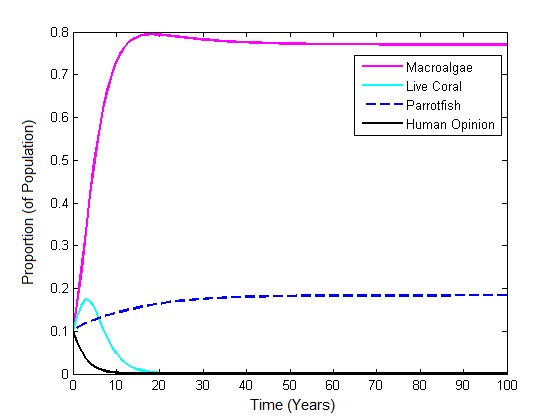


Figure 1: Time series illustrating the behaviour corresponding to *Dead Corals (ii)* for *σ* =0*.*4 *yr*^−1^ and *J* = 1 with corresponding initial conditions *M* = 0*.*1,*C* = 0*.*1,*P* = 0*.*1,*x* = 0*.*1 and all other parameters fixed baseline.


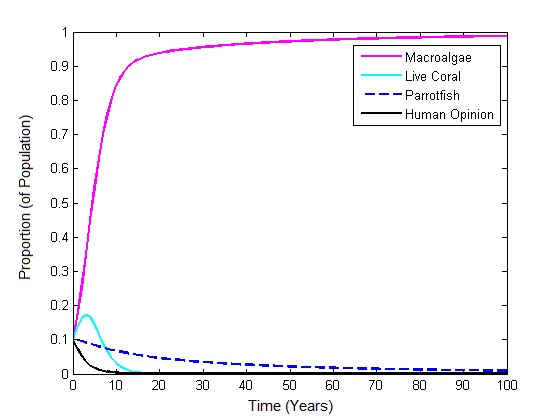


Figure 2: Time series illustrating the behaviour corresponding to *Dead Corals (iii)* for *σ* =0*.*5 *yr*^−1^ and *J* = 1 with corresponding initial conditions *M* = 0*.*1,*C* = 0*.*1,*P* = 0*.*1,*x* = 0*.*1 and all other parameters fixed baseline.


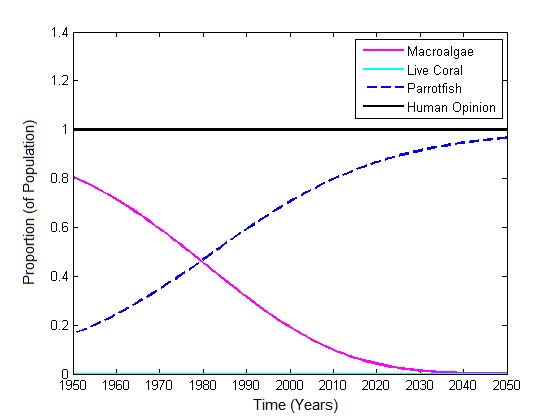


Figure 3: Time series illustrating the behaviour corresponding to *Dead Corals (iv)* for *s* =0*.*4 *yr*^−1^ and *φ* =0*.*6 with corresponding initial conditions *M* =0*.*1,*C* =0*.*9,*P* =0*.*7,*x* =0*.*7 and all other parameters fixed baseline.
